# Determinants of male floating behaviour and floater reproduction in a threatened population of the hihi (*Notiomystis cincta*)

**DOI:** 10.1111/eva.12287

**Published:** 2015-07-28

**Authors:** Patricia Brekke, John G Ewen, Gemma Clucas, Anna W Santure

**Affiliations:** 1 Institute of Zoology, Zoological Society of LondonRegents Park, London, UK; 2 Ocean and Earth Science, National Oceanography Centre Southampton, University of SouthamptonWaterfront Campus European Way, Southampton, UK; 3 Department of Animal and Plant Sciences, University of Sheffield, Western BankSheffield, UK; 4 School of Biological Sciences, University of AucklandAuckland, New Zealand

**Keywords:** Bayesian animal model, heritability, inbreeding, senescence, sexual selection, territorial

## Abstract

Floating males are usually thought of as nonbreeders. However, some floating individuals are able to reproduce through extra-pair copulations. Floater reproductive success can impact breeders’ sex ratio, reproductive variance, multiple paternity and inbreeding, particularly in small populations. Changes in reproductive variance alter the rate of genetic drift and loss of genetic diversity. Therefore, genetic management of threatened species requires an understanding of floater reproduction and determinants of floating behaviour to effectively conserve species. Here, we used a pedigreed, free-living population of the endangered New Zealand hihi (*Notiomystis cincta*) to assess variance in male reproductive success and test the genetic (inbreeding and heritability) and conditional (age and size) factors that influence floater behaviour and reproduction. Floater reproduction is common in this species. However, floater individuals have lower reproductive success and variance in reproductive success than territorial males (total and extra-pair fledglings), so their relative impact on the population's reproductive performance is low. Whether an individual becomes a floater, and if so then how successful they are, is determined mainly by individual age (young and old) and to lesser extents male size (small) and inbreeding level (inbred). Floating males have a small, but important role in population reproduction and persistence of threatened populations.

## Introduction

In many species, competition for mates and territories among males results in some individuals remaining unpaired and without a territory, despite being physically capable of breeding (Smith and Arcese 1989). These individuals are usually defined as ‘floaters’. Floaters may differ from territory holders in age, condition, morphology, behaviour or genetic polymorphisms (Taborsky et al. 2008). Floating has been described in many taxa – fish, birds, mammals and insects (Oliveira et al. 2008), and there is growing realization that floating is important for individual fitness, population regulation and crucially the long-term persistence of populations (Newton 1992; Penteriani et al. 2011; Lenda et al. 2012; Tanferna et al. 2013; Tella et al. 2013).

Floaters can engender persistence through population stability (Franklin 1992; Newton 1992). In particular, floaters can act as buffers or a reservoir against population size changes by rapidly replacing breeders (Grimm et al. 2005), as reserves of genetic diversity (Perrier et al. 2014) and as a warning system against population decline (Franklin 1992; Penteriani et al. 2011). For example, changes in the age composition of breeders due to younger floating individuals entering the breeding population may highlight high levels of adult breeder mortality (Franklin 1992; Penteriani et al. 2011). Importantly, floaters can also gain fertilizations through extra-pair copulations (EPC) and contribute to the next generation (Ewen et al. 1999; Kempenaers et al. 2001).

Floater reproduction can potentially alter the sex ratio among breeders, the variance in reproductive success and the levels of multiple paternity within a population (Smith and Arcese 1989). All of these features of a territorial–floater mating system can influence effective population size *N*_e_ (Nunney 1993; Anthony and Blumstein 2000; Perrier et al. 2014), defined as the size of an ideal population that would lose genetic variability, due to random processes, at the same rate as the actual population (Wright 1931). *N*_e_ determines the rate of inbreeding and genetic drift, which influences the maintenance of genetic variation within threatened populations (Frankham 1995). For example, there is empirical and theoretical evidence that in some species, multiple paternity within broods can decrease reproductive variance and increase *N*_e_ (Sugg and Chesser 1994; Balloux and Lehmann 2003; Pearse and Anderson 2009), while in others, multiple paternity can increase reproductive variance and decrease *N*_e_ (Nunney 1993; Karl 2008), increasing the rate of genetic drift and loss of genetic variation. Any potential reduction in *N*_e_ as a consequence of multiple paternity will be exacerbated in small populations who by their very nature have already lost a proportion of genetic variation through genetic bottleneck events leading to a reduced adaptive potential (Willi et al. 2006).

Paternity gained from EPC would allow floater males, who would not otherwise reproduce, to gain a fraction of the population's reproductive success. This increases the number of breeders, which is particularly important in small populations (Pearse and Anderson 2009). Floaters could also play a role in inbreeding avoidance through extra-pair paternity (Kempenaers et al. 2001; Brekke et al. 2012), increase the genetic diversity within broods (Fiumera et al. 2004) and their presence could result in more equal sharing of paternity among males, leading to a reduction in reproductive variance (Martinez et al. 2000; Hyde et al. 2008). However, floaters may also induce male-biased breeder sex ratio and intensify male–male competition both of which are expected to increase male variance in reproductive success, exacerbating genetic diversity loss (Nunney 1993). Despite the potential importance of floating individuals to conserving genetic variation and population viability, limited empirical evidence is available on floater reproduction and determinants of floater mating behaviour in threatened species (Penteriani et al. 2011; Lenda et al. 2012). This is because monitoring effort is usually directed towards ‘breeding’ individuals (Tella et al. 2013), and in rare species floating individuals can be more elusive and difficult to study (Penteriani et al. 2011). Outside the conservation context, floating as a mating behaviour has been studied intensely (Shuster and Wade 2003; Taborsky et al. 2008; Neff and Svensson 2013) and is known to be determined by one or a mixture of conditional (e.g. age, Arcese 1987; size, Pitnick et al. 2009), environmental (e.g. population density; Bretagnolle et al. 2008; sex ratio, Shuster and Wade 2003) and genetic factors (e.g. inbreeding, Höglund et al. 2002; heritability, Garant et al. 2003).

Floating is particularly common in avian mating systems (Arcese 1987; Smith and Arcese 1989; Newton 1992; Pryke and Andersson 2003; Taborsky et al. 2008; Sergio et al. 2009), including that of many threatened birds (Bretagnolle et al. 2008; Penteriani et al. 2011; Tanferna et al. 2013; Tella et al. 2013). Floating can occur concurrently with other mating behaviours, for example by individuals readily switching from floating to territory holding. Floating behaviour can also change sequentially, for example as individuals grow and age, or can be fixed across an individual's lifetime (Taborsky et al. 2008). In the endemic and endangered passerine the hihi/stitchbird (*Notiomystis cincta*), males can display two mating behaviours: either paired territorial or unpaired floater, they can switch between mating behaviours across their lifetime, but not generally within a season. Both types of male are reproductively mature and engage in solicited and forced EPC (Ewen et al. 1999) resulting in high levels of within-brood multiple paternity (between 1 and 5 sires per brood and ∼70% extra-pair paternity; Brekke et al. 2013). Fitness benefits are not equal; a territorial male's reproductive success, through within-pair and EPC, is nearly three times higher than the EPC reproductive success gained by floating males (Brekke et al. 2012). However, floating males do not incur the costs associated with territory intrusions (e.g. weight loss; Low 2005b) and brood provisioning (up to 32% of feeding visits are by the paired male; Ewen and Armstrong 2000).

The main aims of this study were to estimate floater reproduction and variance in male reproductive success for each mating behaviour and test the genetic and conditional determinants of male floating behaviour and reproductive success in the hihi. Hihi have been intensely monitored since their reintroduction to Tiritiri Matangi Island, New Zealand in 1995 (Brekke et al. 2011). There are a number of advantages of using this island system of a wild, nonmodel species. The population is closed (no emigration/immigration), free-living, and we are able to monitor every individual in the population enabling us to build a detailed pedigree (Brekke et al. 2011). The pedigree is based on long-term monitoring of breeding, banding at the nest, genetic parentage assignment based on 19 microsatellite markers and census data, as well as detailed data on individual reproductive success. This comprehensive data set allows us to reduce the biases and assumptions usually associated with the study of wild territorial–floater systems (Sergio et al. 2009). For example, we are able to monitor all age classes in the population and assess mating behaviour and reproductive contribution across an individual's lifespan. Floaters and nonbreeding territorials can be easily distinguished and territorial and floating males occupy the same habitat making them directly comparable. This makes the hihi system ideal for understanding the evolutionary and conservation implications of floating behaviour.

Previous studies of mating behaviour in birds with and without floating individuals suggest that mating behaviour and reproductive success (rarely tested as floaters assumed to be nonbreeders, but see Kleven et al. 2006; Sardell et al. 2010; Schlicht and Kempenaers 2013 for studies that detected floater reproduction) can depend on four main conditional and/or genetic factors: (i) age, thought to confer skills, experience and motivation to acquire a mate and maintain a territory (e.g. Rohwer et al. 1981; Curio 1983; Shutler and Weatherhead 1991). (ii) Morphology, territorial males are thought to be morphologically superior (e.g. larger or more colourful) than floating males (e.g. Pryke and Andersson 2003). (iii) Inbreeding, thought to depress the ability to acquire a mate, maintain a territory and directly impact reproductive success (e.g. Höglund et al. 2002) and (iv) heritability, with the presence of two behavioural strategies in a population generally maintained by selection (such as frequency dependence or heterozygote advantage) (e.g. Smith and Arcese 1989).

In hihi, male reproductive behaviour (e.g. EPCs, nest provisioning and territory defence) and female reproductive success are known to vary with age (Low et al. 2007; Brekke et al. 2013), intrusion rate (Ewen and Armstrong 2000) and inbreeding level (Brekke et al. 2010). Therefore, based on this and our understanding of other territorial–floater systems, we predict that: (1a) Floater males will be younger and (1b) Have lower age-specific reproductive success than territory holders. (2a) Floater males will be smaller and (2b) Have lower size-specific reproductive success than territory holders. (3a) Floaters are more inbred and (3b) Have lower inbreeding-dependent reproductive success than territory holders. (4) Mating behaviour may be heritable. Testing the genetic (inbreeding and inheritance) and conditional (age and size) factors that determine floating behaviour and male reproductive success would not only inform the conservation management of the population (e.g. if age is a strong predictor of floating behaviour then our predictions of postestablishment growth need to account for the age structure of founders), but also provide evidence for the important determinants of floating behaviour in this threatened population.

## Materials and methods

### Study system

The Tiritiri Matangi Island (36.60°S 174.89°E, in the Hauraki Gulf of New Zealand) population of hihi has been studied and managed intensively as its founding through reintroduction in 1995 (Brekke et al. 2011), including the provision of nest boxes, supplementary feeding and mite control. All individuals fledged are uniquely identifiable throughout their lives with a metal and a combination of coloured bands. Each year two censuses were conducted at the beginning (September) and end (February) of the breeding season (detection probability is relatively high at 0.77, SD = 0.15; Chauvenet et al. 2013). This is a closed population with no immigration or emigration with a growing, male-biased (∼40% F: 60% M) population of ∼150 individuals (Armstrong and Ewen 2013).

### Study species

Hihi are sexually dimorphic and dichromatic, males are larger (∼30%) and brightly coloured. Both sexes can reproduce from their first year of life and can live up to 10 years of age (Low and Pärt 2009). Pairs form at the beginning of the season in September and are generally maintained until the end of the breeding season in February. Pairs can form for one breeding season only or be maintained for several years (Low et al. 2007). Territory holders defend their mate and territory by aggressively displacing intruding males, calling consistently within ∼30 m radius of the nest site and maintaining close proximity to their mate (Low 2005b). Most breeding attempts occurred in nest boxes and were monitored daily. Nest box provision allowed us to follow all breeding events in the population from pair forming, nest building, egg laying to fledging (∼30 days). Females build the nest and incubate the eggs.

### Sampling and parentage assignment

Between 2004 and 2012, blood samples (∼70 *μ*L) were collected from 97% of the banded offspring in the population (1637/1688 from 602 breeding attempts). All individuals were genotyped at 19 highly polymorphic autosomal microsatellite loci (see Brekke et al. 2009 for extraction and amplification details). To reduce genotyping errors (null or false alleles, allelic dropout and stutter), samples were amplified twice, or if not consistent amplified until they were or excluded. Genotyping errors were estimated using Microchecker 2.2.3 (Van Oosterhout et al. 2004). Parentage was assigned to offspring using the maximum-likelihood software Colony 2.0 that incorporated microsatellite data, full- and half-sibship relationships and behavioural information (Wang and Santure 2009). The probability of the true parents being in the candidate lists was set at 0.8, both sexes were defined as polygamous and allele frequencies, and genotyping error rates were provided. The combined exclusion probability of the markers (0.99) used in this study for parental assignment with one known parent was calculated in COANCESTRY v1.0 (Wang 2011). Sires were genetically assigned to 97.5% of the sampled offspring with >95% confidence providing an accurate record of male reproductive success (for details see Brekke et al. 2012).

### Pedigree building

Behavioural information on each breeding event was used to link the dam to the offspring banded from founding in 1995 to February 2012 (*n* = 2083 assigned out of 2098 fledged; 99.3% coverage). Dams identified from social behaviour were correctly assigned genetically 99.2% of the time. As behavioural information on egg laying was available from founding, the maternal line was retained prior to genetic data being available, to maximize the information on maternal half- and full-sibs. Whole population genetic sampling was initiated in 2004 (some individuals were sampled in previous years) and was used to add paternity links from 2001 to 2012 in the pedigree (*n* = 1399 assigned out of 2098 fledged; 66.7% coverage). The pedigree was used to calculate inbreeding coefficients (*f*) for all males with four known grandparents (*n* = 159). This may bias sampling towards shorter-lived individuals (Table S1). However, very few individuals survive longer than 7 years of age (20 of 830 observations; Table S1). Inbreeding coefficients are sensitive to pedigree depth, completeness and the baseline population, which in this case is represented by the 21 individual founders of the Tiritiri Matangi population, who were assumed to be unrelated (Brekke et al. 2011).

### Determinants of floating behaviour

To establish whether age (in years, linear and quadratic; prediction 1a), size (prediction 2a) or individual inbreeding coefficient (*f*) (prediction 3a) determined whether a male became a territory holder or floater within each breeding season, we fitted generalized linear mixed models (GLMMs), evaluated with maximum likelihood, with a binomial response variable (territorial/floater) and a logit-link function. We fitted fixed factors of age (both linear and quadratic; prediction 1a), size (prediction 2a) and inbreeding (prediction 3a) and included interactions between inbreeding and (i) age and (ii) size to test whether inbreeding depressed these measures of male quality and reduced the likelihood of a male becoming territorial. Size was determined by tarsus length measured at 21 days of age, as this morphological trait remains unchanged from this stage (Low 2006). Unfortunately, no other information on annual male size or potentially sexually selected traits was available. The models additionally fitted random factors for year and individual to account for nonindependence among multiple observations (361 observations for 161 males, 83 of which bred more than once; see Table S2), especially among longer lived individuals. Model selection for all analyses was performed using Akaike's information criterion (AIC; Burnham and Anderson 1998) and model-averaged coefficients were generated by averaging across models with ΔAICs <2 using the package MuMIn following Grueber et al. (2011) in the R statistical programming environment (R Development Core Team 2012). All explanatory variables were standardized (mean = 0, variance = 1), which is necessary for model averaging. The parameters with the highest relative importance were incorporated into models for repeatability and heritability described below.

### Repeatability and heritability of floating behaviour

Individual repeatability (*R*) is defined as the proportion of phenotypic variation that is reproducible among repeated measurements of the same subject or group (Lessells and Boag 1987) and can be used to quantify the extent to which an individual's behaviour remains consistent over time. Repeatability may include both genetic and environmental sources of variation. The narrow-sense heritability (*h*^2^) of a trait is the proportion of phenotypic variance due to additive genetic variance (Boake 1989; Falconer and Mackay 1996; Lynch and Walsh 1998), while environments that affect individuals in a constant manner across repeated measures of the same individual are termed permanent environment (PE) effects (Kruuk and Hadfield 2007). To estimate the contribution of repeatability, heritability and PE effects to the variance in mating behaviour (prediction 4a and 4b), we ran two GLMMs to partition the contribution of these terms to the overall phenotypic variance (*V*_P_). In both models, we classified male mating behaviour per season as floater or territorial and accounted for the fixed effects of overall intercept and age (linear and quadratic) and additionally fit year as a random effect. The repeatability (model 2a) can be estimated in a mixed model framework by fitting the individual identity as a random effect; *R* = *V*_R_/*V*_P_ where *V*_R_ is the repeatability variance. Heritability and PE effects are jointly estimated in a second mixed model (model 2b). The additive genetic variance (*V*_A_) is estimated by fitting the relatedness between individuals (as estimated from their pedigree relationships) as a random effect, with heritability calculated as *h*^2^ = *V*_A_/*V*_P_. The variance due to PE (*V*_PE_) is estimated by fitting individual identity as a random effect.

Information for all fixed and random effects and mating status were available for 830 records, which represented 289 males, 171 of which bred over more than one season.

Variance components were estimated using Bayesian Markov chain Monte Carlo (MCMC) in the R (R Development Core Team 2012) package MCMCglmm (Hadfield 2010). Mating status was classified as a categorical variable with two levels (floater and territorial). Trialling a number of different priors produced similar variance component estimates, with low autocorrelation among iterations in most runs. We present results for priors where all variances were set to 1, with a degree of belief of 1; these priors were chosen as they gave consistent estimates for variance components across trial runs. Model 2a was run for 1 000 000 iterations with a burn-in of 50 000 iterations and estimates stored every 500 iterations, while model 2b was run for 1 500 000 iterations with a burn-in of 50 000 iterations and estimates stored every 700 iterations to achieve convergence. Autocorrelation between iterations was low (<0.05 for all variance and covariance components in both models). Fitting mating status as a categorical variable requires a logit-link function; therefore, the overall phenotypic variance (*V*_P_) is


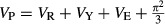


for model 2a and


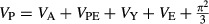


for model 2b, where 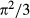 is the variance for the logistic distribution (Nakagawa and Schielzeth 2010). In addition to estimating variance components contributed from each random effect, the support for the contribution of additive genetic, PE and year to overall variance was assessed using DIC to compare models constructed with and without these variance components.

### Reproductive variance

We quantified male annual reproductive success (ARS) for territorial (which includes within-pair and extra-pair reproduction) and floating (extra-pair reproduction only) males by estimating the numbers of offspring sired and fledged each year. The distribution of ARS for each mating behaviour was described using the mean, interquartile range (IQR) and the proportion of males with zero reproductive success. We also calculated three measures of variation in mating behaviour-specific ARS: (i) the variance (Var, the second moment of the distribution) in ARS. (ii) The maximum opportunity for selection (*I*) which is the standardized mean variance in ARS and describes the distribution of reproductive success within each class (Arnold and Wade 1984) and (iii) Morisita's index (*I*_*δ*_) (Morisita 1962), a predictor of spatial clumping.

### Reproductive success

**Figure 1:**
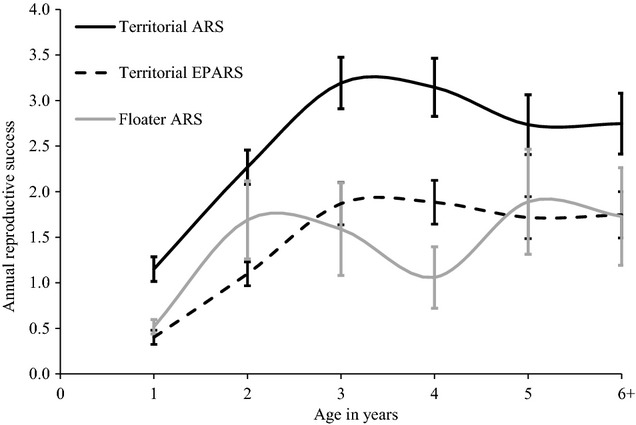
Raw data showing territorial and floater male age-specific reproductive success. The black solid line refers to territorial males’ mean annual reproductive success (ARS), the broken black line refers to territorial males’ mean extra-pair annual reproductive success (EPARS), and grey solid line refers to floater males mean ARS. Standard error bars are shown for all raw values. Territorial males’ sample sizes (age 1 = 107; age 2 = 123; age 3 = 99; age 4 = 69; age 5 = 49; age 6+ = 67). Floater males’ samples sizes (age 1 = 146; age 2 = 29; age 3 = 17; age 4 = 17; age 18 = 6; age 6+ = 22).

We tested whether ARS and extra-pair annual reproductive success (EPARS; total number of extra-pair offspring fledged) were predicted by male mating behaviour (predictions 1b, 2b and 3b), age (in years, linear or quadratic) (prediction 1b), size (tarsus length) (prediction 2b) or individual inbreeding coefficient (*f*) (prediction 3b) (see Table S2 for sample sizes). We modelled age as both a linear and quadratic variable, reflecting the expected linear or ‘humped’ relationships between age and fitness (e.g. Low et al. 2007; Brekke et al. 2013). We used GLMMs, evaluated with maximum likelihood, with Poisson error structure and a log-link function. Models fitted year and individual identity (358 observations for 159 males, 82 of which bred more than once) as random effects. We also tested interactions between mating behaviour and (i) inbreeding, (ii) age and (iii) tarsus length to check whether these measures of male quality explained differences in reproductive success between floater and territorial males (predictions 1b, 2b, 3b) (Model set 3). In addition, we show raw averages of age-specific variation in ARS and EPARS for territory holders and floaters, not subject to statistical analysis, but to substantiate patterns in observed ARS and EPARS (Fig. 1).

## Results

### Determinants of floating behaviour

**Figure 2:**
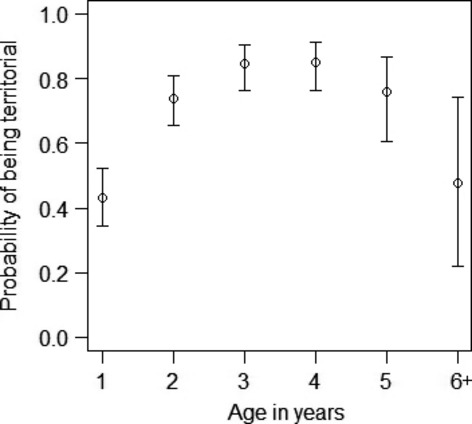
Changes in the probability of a male becoming territorial with age. Vertical lines are the 95% confidence intervals around the mean.

On average, a third of males in the population became floaters (Table S3). Male mating behaviour was also strongly age dependent (Fig. 2). The relationship between age and mating status was quadratic, with an increase in the likelihood of becoming a territorial between the ages of one and two and a decline in males over five (Fig. 2; Table 1; Tables S1 and S4). However, we note that relatively few males survive and reproduce past the age of 5 years. Inbreeding and tarsus length were included in the top-model set and averaged model and had a relatively high importance, but had no significant effect on whether a male became a floater or territory holder (Table 1; Table S4). None of the interactions tested were included in the top-model set or had a significant effect on male mating status (Table S4).

**Table 1:** Parameter estimates for each of the top models (AICc <2) in the confidence set for male annual mating behaviour (AMB) (Model set 1). Models are ranked by AICc, for each model the number of parameters (*k*), AICc, delta AICc (ΔAICc) and Akaike weight (A*i*) are provided. Below the model-averaged estimates are provided with their confidence intervals (CI) and relative importance. In bold are the parameters with significant (*P* < 0.001) effect on male mating behaviour. *Age* and *Age2* refer to linear and quadratic age functions respectively, *f* to inbreeding and *Tarsus* to male tarsus length. None of the top models included interactions (Age2: *f*; Age2: Tarsus or Tarsus: *f*)

AMB models	Intercept	Age	Age2	*f*	Tarsus	*k*	AICc	ΔAICc	A*i*
Age + Age2 + Tarsus	1.158	**2.433**	**−2.187**		0.469	6	444.9	0.00	0.26
Age + Age2	1.092	**2.291**	**−2.158**			5	445.6	0.68	0.19
Age + Age2 + *f* + Tarsus	1.156	**2.450**	**−2.188**	−0.353	0.468	7	445.7	0.77	0.18
Age + Age2 + *f*	1.080	**2.300**	**−2.172**	−0.364		6	446.2	1.27	0.14
Model-average est.	1.127	**2.378**	**−2.178**	−0.358	0.468				
CI 2.5%	0.717	1.608	−3.051	−0.942	−0.035				
CI 97.5%	1.537	3.148	−1.304	0.227	0.972				
Relative importance		1.00	1.00	0.41	0.58				

### Heritability of floating behaviour

Mating behaviour was repeatable over an individual's lifetime (0.148, credible interval 0.060–0.316, Table S5), suggesting that mating status was relatively consistent over a male's lifetime. Given the repeatability of mating status, we also tested whether it was heritable. The estimates for the contribution of the additive genetic and year effects to overall phenotypic variance in mating status were very low and not significant, while the contribution of PE to overall variance was moderate (Table 2). The estimated heritability (proportion of variance explained by the additive genetic effect) was 0.001 (Table 2). Furthermore, DIC values for models constructed without additive genetic effects and without year effects suggest weak or no support for including these terms in the full model. In contrast, the estimate for the contribution of PE effects to variance in mating status (0.106) was well supported by comparison of the DIC values for a model without this term (Table 2).

**Table 2:** Estimates and proportion of variance explained for the contribution of additive genetic, permanent environment and year to overall variance in mating behaviour, with 95% credible intervals (CI) (Model 2a). ΔDIC is calculated as the DIC for the full model (939.661) minus DIC for a model without the random effect; large negative numbers indicate strong support for keeping the term in the model

Random effect	Estimate (CI)	Proportion of variance explained (CI)	DIC (model without this term)	ΔDIC
Additive genetic	0.008 (0.000, 1.003)	0.001 (0.000, 0.179)	939.479	0.182
Permanent environment	0.574 (0.000, 1.570)	0.106 (0.000, 0.260)	949.155	−9.49
Year	0.003 (0.000, 0.264)	0.000 (0.000, 0.042)	939.898	−0.24

### Reproductive variance

Floater and territorial males differed substantially in all descriptors of reproductive success (median, IQR, proportion with zero ARS, mean; Table 3). As a consequence of the low mean ARS for floaters, floater males have a lower variance in ARS than territorial males. However, the maximum opportunity for selection (*I*), measured as the standardized variance in ARS, and Morisita's index, a measure of how uniformly fitness is distributed across individuals (*I*_*δ*_), were much higher in floating males than territorial males. Unfortunately, it is not possible to test directly whether floaters in the population increase or decrease overall reproductive variance, as any reallocation of offspring from floater males to territorial males (to test the impact that floaters had on overall variation) would change the mean ARS (which in itself would impact the population variance) but would also remove important effects of competition between territorial males in their own within-pair and extra-pair matings. However, once standardized by the small overall reproductive success of floaters, the results from Table 3 suggest that the standardized variance in ARS for floating individuals is higher than that of territorials and that there is a larger difference in reproductive success within floaters than within territorials.

**Table 3:** Statistics describing all males, territorial and floating male's annual reproductive success (ARS). Med refers to median; IQR refers to interquartile range; Prop. Zeros refers to the proportion of males with zero reproduction in each class; *µ* refers to the mean ARS; Var(ARS) refers is the variance in male ARS; *I*(ARS) refers to the maximum opportunity for selection and *Iδ*(ARS) refers to Morisita's index

	Obs	n	Med	IQR	Prop. zeros	*μ*	Var (ARS)	*I* (ARS)	*I*δ (ARS)
All males	764	283	1	0–3	0.39	1.96	5.33	1.39	1.88
Territorial males	514	196	2	0–4	0.27	2.44	5.86	0.99	1.58
Floating males	250	87	0	0–1	0.62	0.97	2.77	2.96	2.94

### Reproductive success

**Figure 3:**
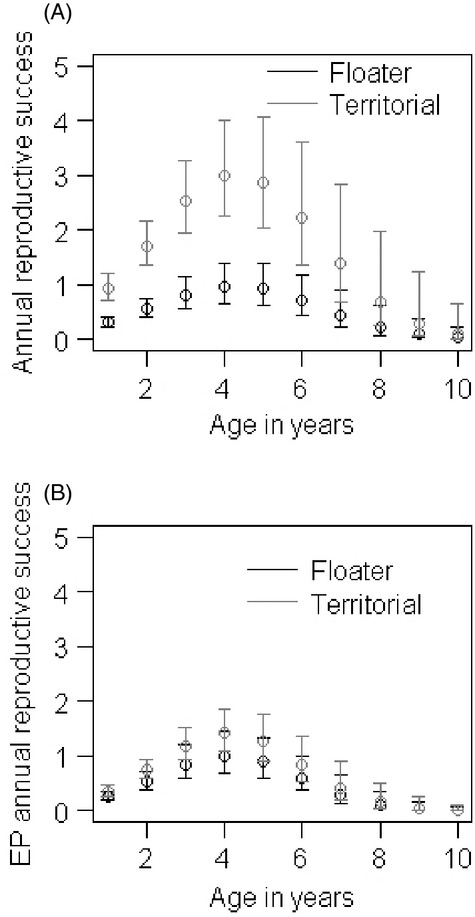
Generalized linear mixed model (GLMM) plots based on the average model parameters (Model set 3) showing changes in (A) annual reproductive success (ARS) with age and (B) extra-pair (EP) ARS with age for territorial (dark grey solid lines) and floater (black solid line) males. Vertical lines are the 95% confidence intervals around the mean.

We found a strong quadratic, age-dependent male ARS and EPARS for territorial males and EPARS for floaters, with first-year and over 5-year olds having lower reproductive success relative to males in their prime (Fig. 3A; Table 4; Table S6 and S7). However, we have relatively few observations for males 5 years or older. Territorial males have a much higher within-pair and extra-pair reproductive success across their lifetime, despite floater males ‘specializing’ in EPCs (Fig. 3B; Table 4a,b). Inbreeding and tarsus length were both included in the top models that explained ARS and EPARS (Table 4a,b), but were not significant. None of the interactions tested were included in the top-model set or had a significant effect on male reproductive success.

**Table 4:** Parameter estimates for each of the top models (AICc <2) in the confidence set for male (a) annual reproductive success (ARS) and (b) extra-pair annual reproductive success (EPARS) (Model set 3). Models are ranked by AICc, for each model the number of parameters (*k*), AICc, delta AICc (ΔAICc) and Akaike weight (A*i*) are provided. Below the model-averaged estimates are provided with their confidence intervals (CI) and relative importance. In bold are the parameters with significant (*P* < 0.001) effect on male reproductive success. *Age* and *Age2* refer to linear and quadratic age functions respectively, *Behaviour* to male mating behaviour, *f* to inbreeding and *Tarsus* to male tarsus length. None of the top models included an interaction (Behaviour: *f*)

ARS models	Intercept	Behaviour	Age	Age2	*f*	Tarsus	*k*	AICc	ΔAIC_c_	A*i*
(a)										
Age + Age2 + Behaviour	0.192	**1.150**	**1.299**	**−0.881**			6	603.2	0.00	0.346
Age + Age2 + Behaviour + *f*	0.187	**1.142**	**1.301**	**−0.880**	−0.250		7	603.7	0.44	0.278
Age + Age2 + Behaviour + Tarsus	0.196	**1.147**	**1.312**	**−0.884**		0.058	7	605.2	1.97	0.129
Model-average est.	0.191	**1.146**	**1.302**	**−0.881**	−0.250	0.058				
CI 2.5%	−0.044	0.857	0.981	−1.212	−0.637	−0.280				
CI 97.5%	0.426	1.436	1.623	−0.550	0.138	0.397				
Relative importance		1.00	1.00	1.00	0.37	0.17				

EPARS models	Intercept	Behaviour	Age	Age2	*f*	Tarsus	*k*	AICc	AAICc	A*i*
(b)												
Age + Age2 + Behaviour	−0.321	**0.343**	**1.614**	**−1.204**			6	545.0	0.00	0.307
Age + Age2 + Behaviour + *f*	−0.329	**0.335**	**1.620**	**−1.202**	−0.253		7	545.9	0.94	0.192
Age + Age2 + Behaviour + Tarsus	−0.330	**0.348**	**1.598**	**−1.190**		−0.115	7	546.7	1.79	0.125
Model-average est.	−0.325	**0.341**	**1.613**	**−1.201**	−0.253	−0.115				
CI 2.5%	−0.543	0.021	1.200	−1.620	−0.735	−0.552				
CI 97.5%	−0.107	0.661	2.025	−0.782	0.229	0.323				
Relative importance		1.00	1.00	1.00	0.31	0.20				

## Discussion

Here, we have shown that floating males have a small, but important role in population reproduction, by increasing the number of breeders. Our study mainly supports our age-specific predictions. Age is the strongest determinant of floating behaviour and male reproductive success. Males float when they are young (1 year) or old (over 5 years) and the ARS of floating males is lower than the ARS and EPARS of territorial males. The heritability and inbreeding predictions were not supported as male mating behaviour did not have a significant genetic basis. Mating behaviour lacked additive genetic variance, that is was not heritable (close to zero with large CI) and had a high PE component. These patterns of conditional-dependent mating behaviour appear to maximize male fitness for each behavioural type. Below, we discuss each finding and review them within a conservation context.

Floaters could be a potentially important genetic pool of individuals for hihi populations. Hihi floaters can reproduce through extra-pair paternity, increasing the number of breeders and contributing to the population's reproductive output. Floater reproduction may not be evident, as in many studies all males are rarely sampled. However, when a large proportion of unpaired/floater males are sampled, they are found to gain a reasonable proportion of extra-pair paternity reproduction (Kleven et al. 2006; Sardell et al. 2010). Extra-pair paternity can change the variance in male reproductive success by reassigning the distribution of paternity across the population (Nunney 1993). In hihi, the variance in extra-pair reproduction for territorial males has the largest contribution to male reproductive success (60% of fertilizations; Walker et al. 2014). Therefore, studies that do not sample floating/unpaired males may misinterpret the effect of extra-pair paternity on variance in reproductive success (Shuster 2009). Unfortunately, given the large difference in mean reproductive success between floaters and territorials, we could not establish directly the impact of floater reproduction on the overall variance in reproductive success. Regardless, in small populations, floater reproduction is likely to have a positive effect, as it increases the total number of males breeding each season, and floater's genetic contribution to future generations will have the general effect of decreasing inbreeding.

The reproductive contribution of floaters also varies across their lifetime. Floater reproduction follows the same dome-shape distribution seen in territorial males (within-pair and extra-pair, this study) and females (overall; Low and Pärt 2009; extra-pair, Brekke et al. 2013) and contrasts the u-shaped social male age-specific cuckoldry patterns (Brekke et al. 2013). Hihi reproduction and mating behaviour is strongly age-structured and shows signs of senescence. First-year male hihi, as seen in most studies of territorial–floater systems are likely to be floaters (Smith and Arcese 1989; Sergio et al. 2009). However, unlike most studies, we have also shown older males (over 5 years) tend to also become floaters and there is likely to be senescence in floater extra-pair reproduction. Therefore, the contribution of floater males to the population's reproductive rate is likely to be higher for middle-aged males (2–4 years of age) than young (1 year old) or old (post 5 years of age) floater males. This age-specific contribution to reproduction is likely to impact the age structure of the population, demographic changes in population size, effective population size and rate of genetic drift (Engen et al. 2005).

The dome-shaped age-related patterns in male reproductive success and mating behaviour shown here have been found in a number of species (Age-specific reproductive success – Forslund and Pärt 1995; Keller et al. 2008; Lebigre et al. 2013) (Age-determined mating behaviour – Smith and Arcese 1989; Shutler and Weatherhead 1991; Newton and Rothery 2001; Sergio et al. 2009; Penteriani et al. 2011). They are usually associated with poor quality individuals dying young, unable to fight for a territory and having low reproductive success, and survivors having improved skills and reproductive output (Forslund and Pärt 1995). Young individuals may have lower reproductive success as they are inexperienced. Inexperience can impact male–male competition for territories (Low 2005a), mating, particularly as mating in this species is frequently forced (Brekke et al. 2013) and experience of the landscape. Competition for food resources is less likely to be an important factor as this population is supplementary fed (Chauvenet et al. 2013). Middle-aged individuals are likely to become more dominant and fight harder for territories and females as they have lower residual reproductive value than young individuals. However, this trade-off between territoriality and reproductive success may become unsustainable for older individuals. Middle-aged territorial males may also be preferred by females as social and extra-pair partners, as they can offer paternal care and lower risk of forced copulation (Low et al. 2007; Brekke et al. 2013).

Mating behaviour in hihi had very low *V*_A_. However, the mixed model analysis indicates that the behaviour of individuals is strongly repeatable over their lifetime (as indicated by the large PE effect). The lack of heritability in mating behaviour may not only be due to a strong environmental variance component, but also lack of power from the difficulties arising from applying animal models to wild populations (Kruuk et al. 2002). Lack of *h*^2^ may also be due to allelic fixation or if genetic drift has eroded *V*_A_ in this small, reintroduced population. Regardless, behavioural traits, closely linked to fitness and under strong directional selection, such as courtship displays (Hedrick 1994) and extra-pair reproductive success (Reid et al. 2011) are generally expected to have low *V*_A_ and high *V*_D_ (Mousseau and Roff 1987). Low *h*^2^ would suggest there are limited indirect genetic benefits for females mating with territorial males. However, this does not exclude the possibility that male size or territories themselves may be inherited or that phenotypic plasticity in mating behaviour has a strong genetic basis in this species and remains to be explicitly tested.

A large proportion of conservation-based studies place most of the emphasis on understanding the dynamics of breeding individuals and most management effort is directed towards them. But this study highlights the importance of not making a-priori assumptions about unpaired, floating individuals. We have demonstrated that floating individuals, often assumed to sire few or no offspring, do reproduce and contribute to the total reproductive variance in the population. Whether their impact has a positive or negative effect on population demographic processes will depend on the trade-off between their genetic contribution and the impact of sexual conflict in male-biased populations. In hihi, it appears there is probably a positive effect as cost of sexual conflict on demography is small (Ewen et al. 2011) and floaters increase the number of breeders.

## Data archiving statement

Data available from the Dryad Digital Repository: http://dx.doi.org/10.5061/dryad.f06t6.

## Supporting Information

**Table S1.** Male sample sizes per variable from 2004 to 2012 breeding seasons. **Table S2.** Sample sizes for AMT and ARS/EPARS analysis for males with four known grandparents, tarsus length, age and reproductive success information. **Table S3.** Proportion of floater males in the population per year. **Table S4.** Mating behaviour candidate model-set. Top models in bold (ΔAIC <2). **Table S5.** Estimates and proportion of variance explained for the contribution of individual repeatability and year to overall variance in mating behaviour, with 95% credible intervals (CI). **Table S6.** Annual reproductive success candidate model-set. Top models in bold (ΔAIC <2). **Table S7.** Annual extra-pair reproductive success candidate model-set. Top models in bold (ΔAIC <2).
