# Supplementary material for: Determinants of male floating behaviour and floater reproduction in a threatened population of the hihi (Notiomystis cincta)
Source: Evol Appl. 2015 Jul 28;8(8):796–806. doi: 10.1111/eva.12287 (PMC4561569; doi:10.1111/eva.12287)
Supplement: Supplementary file 1 [file eva0008-0796-sd1.docx]

**Supplementary material**

**Table S1** Male sample sizes per variable from 2004-2012 breeding seasons.

| Age | Territorial | Floater | Territorial with ARS/EPARS | Floater with ARS | Territorial with tarsus | Floater with Tarsus | Territorial with *f* | Floater with *f* |
| --- | --- | --- | --- | --- | --- | --- | --- | --- |
| 1 | 124 | 165 | 107 | 146 | 124 | 165 | 71 | 91 |
| 2 | 138 | 33 | 123 | 29 | 138 | 33 | 66 | 17 |
| 3 | 107 | 18 | 99 | 17 | 107 | 18 | 41 | 12 |
| 4 | 71 | 17 | 69 | 17 | 71 | 17 | 26 | 7 |
| 5 | 49 | 18 | 49 | 18 | 49 | 18 | 13 | 6 |
| 6 | 32 | 9 | 32 | 8 | 32 | 9 | 5 | 3 |
| 7 | 20 | 9 | 20 | 9 | 20 | 9 | 2 | 2 |
| 8 | 11 | 3 | 11 | 3 | 11 | 3 | NA | NA |
| 9 | 3 | 1 | 3 | 1 | 3 | 1 | NA | NA |
| 10 | 1 | 1 | 1 | 1 | 1 | 1 | NA | NA |

**Table S2** Sample sizes for AMT and ARS/EPARS analysis for males with four known grandparents, tarsus length, age and reproductive success information.

|  | Mating behaviour | | ARS/EPARS | |
| --- | --- | --- | --- | --- |
| Year | Territorial | Floater | Territorial | Floater |
| 2005 | 1 | 3 | 1 | 2 |
| 2006 | 6 | 9 | 6 | 9 |
| 2007 | 10 | 18 | 9 | 18 |
| 2008 | 23 | 16 | 23 | 16 |
| 2009 | 36 | 24 | 36 | 24 |
| 2010 | 44 | 22 | 44 | 22 |
| 2011 | 46 | 20 | 46 | 19 |
| 2012 | 57 | 26 | 57 | 26 |

**Table S3** Proportion of floater males in the population per year.

| Year | Total No of males | Floater | Territorial | Proportion of floaters in the population |
| --- | --- | --- | --- | --- |
| 2004 | 38 | 14 | 24 | 0.37 |
| 2005 | 74 | 39 | 35 | 0.53 |
| 2006 | 80 | 26 | 54 | 0.33 |
| 2007 | 94 | 26 | 68 | 0.28 |
| 2008 | 93 | 29 | 64 | 0.31 |
| 2009 | 102 | 32 | 70 | 0.31 |
| 2010 | 100 | 31 | 69 | 0.31 |
| 2011 | 89 | 26 | 63 | 0.29 |
| 2012 | 105 | 34 | 71 | 0.32 |

**Table S4** Mating behaviour candidate model-set. Top models in bold (ΔAIC < 2).

| Model selection table | |  |  |
| --- | --- | --- | --- |
| Model number | AICc | ΔAICc | A*i* |
| **1** | **444.9** | **0** | **0.262** |
| **2** | **445.6** | **0.68** | **0.186** |
| **3** | **445.7** | **0.77** | **0.178** |
| **4** | **446.2** | **1.27** | **0.139** |
| 5 | 447.6 | 2.67 | 0.069 |
| 6 | 448 | 3.08 | 0.056 |
| 7 | 448.8 | 3.89 | 0.037 |
| 8 | 449.8 | 4.84 | 0.023 |
| 9 | 450.3 | 5.32 | 0.018 |
| 10 | 450.8 | 5.86 | 0.014 |
| 11 | 451.7 | 6.73 | 0.009 |
| 12 | 453 | 8.02 | 0.005 |
| 13 | 454.9 | 9.98 | 0.002 |
| 14 | 457.5 | 12.55 | 0 |
| 15 | 478.1 | 33.12 | 0 |
| 16 | 478.9 | 33.97 | 0 |
| 17 | 479.8 | 34.88 | 0 |
| 18 | 480.7 | 35.79 | 0 |
| 19 | 482.3 | 37.37 | 0 |

**Table S5** Estimates and proportion of variance explained for the contribution of individual repeatability and year to overall variance in mating behaviour, with 95% credible intervals (CI). ΔDIC is calculated as the DIC for the full model (939.179) minus DIC for a model without the random effect; large negative numbers indicate strong support for keeping the term in the model.

| Random effect | Estimate (CI) | Proportion of variance  explained (CI) | DIC (model without this term) | ∆DIC |
| --- | --- | --- | --- | --- |
| Repeatability | 0.752 (0.255, 1.992) | 0.148 (0.060, 0.316) | 966.438 | -27.259 |
| Year | 0.001 (0.000, 0.253) | 0.000 (0.000, 0.045) | 939.649 | -0.470 |

**Table S6** Annual reproductive success candidate model-set. Top models in bold (ΔAIC < 2).

| Model selection table | |  |  |
| --- | --- | --- | --- |
| Model number | AICc | ΔAICc | A*i* |
| **1** | **737.5** | **0** | **0.443** |
| **2** | **739.1** | **1.53** | **0.206** |
| **3** | **739.4** | **1.91** | **0.171** |
| 4 | 741 | 3.43 | 0.08 |
| 5 | 741.1 | 3.62 | 0.072 |
| 6 | 743.1 | 5.53 | 0.028 |
| 7 | 808.6 | 71.12 | 0 |
| 8 | 808.7 | 71.23 | 0 |
| 9 | 810.3 | 72.76 | 0 |
| 10 | 810.4 | 72.86 | 0 |
| 11 | 812.4 | 74.84 | 0 |
| 12 | 812.4 | 74.92 | 0 |
| 13 | 833 | 95.5 | 0 |
| 14 | 833.8 | 96.23 | 0 |
| 15 | 834.7 | 97.17 | 0 |
| 16 | 835.4 | 97.9 | 0 |
| 17 | 939.4 | 201.84 | 0 |
| 18 | 939.6 | 202.1 | 0 |
| 19 | 940.2 | 202.66 | 0 |
| 20 | 940.4 | 202.89 | 0 |
|  |  |  |  |
|  |  |  |  |

**Table S7** Annual extra-pair reproductive success candidate model-set. Top models in bold (ΔAIC < 2).

| Model selection table |  |  |  |
| --- | --- | --- | --- |
| Model number | AICc | ΔAICc | A*i* |
| **1** | **545** | **0** | **0.307** |
| **2** | **545.9** | **0.94** | **0.192** |
| **3** | **546.7** | **1.79** | **0.125** |
| 4 | 547.6 | 2.68 | 0.081 |
| 5 | 547.7 | 2.77 | 0.077 |
| 6 | 547.7 | 2.78 | 0.077 |
| 7 | 548.3 | 3.36 | 0.057 |
| 8 | 549.5 | 4.58 | 0.031 |
| 9 | 549.6 | 4.6 | 0.031 |
| 10 | 550.2 | 5.29 | 0.022 |
| 11 | 605.8 | 60.83 | 0 |
| 12 | 606.5 | 61.59 | 0 |
| 13 | 607.1 | 62.11 | 0 |
| 14 | 607.8 | 62.83 | 0 |
| 15 | 609.1 | 64.18 | 0 |
| 16 | 609.9 | 64.9 | 0 |
| 17 | 626.6 | 81.62 | 0 |
| 18 | 626.8 | 81.87 | 0 |
| 19 | 627.5 | 82.52 | 0 |
| 20 | 627.7 | 82.77 | 0 |
